# International Standards for Neurological Classification of Spinal Cord Injury: Classification Questions and Cases

**DOI:** 10.46292/sci25-00013

**Published:** 2025-08-22

**Authors:** Brittany Snider, Steven Kirshblum, Ruediger Rupp, Christian Schuld, Fin Biering-Sorensen, Stephen Burns, James Guest, Linda Jones, Andrei Krassioukov, Gianna Rodriguez, Mary Schmidt Read, Keith Tansey, Kristen Walden

**Affiliations:** 1 Kessler Institute for Rehabilitation, West Orange, New Jersey, USA; 2 Rutgers New Jersey Medical School, Department of Physical Medicine & Rehabilitation, Newark, New Jersey, USA; 3 Spinal Cord Injury Center, Heidelberg University Hospital, Heidelberg, Germany; 4 Medical Faculty Heidelberg, Heidelberg University, Heidelberg, Germany; 5 Department of Clinical Medicine, University of Copenhagen, and Department of Brain and Spinal Cord Injuries, Rigshospitalet, Copenhagen, Denmark; 6 Department of Rehabilitation Medicine, University of Washington School of Medicine, Seattle, Washington, USA; 7 University of Miami, Miller School of Medicine, Department of Neurological Surgery, Miami, Florida, USA; 8 Thomas Jefferson University, Philadelphia, Pennsylvania, USA; 9 International Collaboration on Repair Discovery (ICORD), University of British Columbia, Vancouver, British Columbia, Canada; 10 Michigan Medicine, Department of Physical Medicine and Rehabilitation, Ann Arbor, Michigan, USA; 11 Magee Rehabilitation Hospital, Jefferson Health, Philadelphia, Pennsylvania, USA; 12 Center for Neuroscience and Neurological Recovery, Methodist Rehabilitation Center, Jackson, Mississippi, USA; 13 Spinal Cord Injury Medicine and Research Services, Jackson Veterans Administration Medical Center, Jackson, Mississippi, USA; 14 Department of Neurosurgery, University of Mississippi Medical Center, Jackson, Mississippi, USA; 15 Praxis Spinal Cord Institute, Vancouver, Canada

**Keywords:** classification accuracy, International Standards for Neurological Classification of Spinal Cord Injury (ISNCSCI), nontraumatic SCI, spinal cord injury (SCI), spinal cord stimulation, tendon transfer

## Abstract

**Background:**

The International Standards for Neurological Classification of Spinal Cord Injury (ISNCSCI) have been refined through the years and continue to evolve with advances in the field. The International Standards Committee of the American Spinal Injury Association (ASIA) is responsible for maintaining, continually reviewing, and updating the ISNCSCI. Questions from spinal cord injury (SCI) professionals are frequently submitted to ASIA for review by the International Standards Committee.

**Methods:**

Of the questions submitted to the International Standards Committee, 5 were selected for this article, as they relate to common areas of confusion, address challenging classification concepts, and have not previously been described. Representative cases were also created to reinforce classification rules and the committee's recommendations.

**Cases:**

The 5 questions/cases address ISNCSCI classification in the setting of (1) AIS E grade, (2) tendon transfer, (3) spinal cord stimulation, (4) nontraumatic SCI (ntSCI) etiology, and (5) AIS D grade (vs. AIS B) based on the presence of non-key muscle function. Each case includes a detailed review of the correct classification components and thorough discussion of the impact the corresponding question has on the classification.

**Conclusion:**

The International Standards Committee provides answers to questions about ISNCSCI classification. The scenarios presented in this article address important classification rules and challenging concepts that have not previously been described. This article can serve as a useful reference when similar cases are encountered in clinical and research settings.

## Introduction

Initially published in 1982,^1^ the International Standards for Neurological Classification of Spinal Cord Injury (ISNCSCI) have been refined through the years and continue to evolve with advances in the field, accruing knowledge, technological innovations, and emerging data. The most recent ISNCSCI edition was published in 2019,^2^ with reference articles^3^–^5^ to explain the revisions.

The International Standards Committee of the American Spinal Injury Association (ASIA) is responsible for maintaining, continually reviewing, and updating the ISNCSCI and associated documents and resources. Questions from spinal cord injury (SCI) professionals on the ISNCSCI examination and classification are frequently received and reviewed by the International Standards Committee and have been addressed in peer-reviewed publications.^6^,^7^ These questions and feedback from international SCI clinicians and researchers often influence ISNCSCI revisions and clarifications.

This article and its complementary article,^8^ which focuses on the 2019 revision, include cases from the ISNCSCI workbook^9^ and are part of a wider educational initiative to enhance understanding of ISNCSCI classification.^5^,^7^,^10^–^12^ In this article, the International Standards Committee addresses questions and specific scenarios, such as accurate ISNCSCI classification in the setting of a tendon transfer or spinal cord stimulation, which have not previously been described.

## Methods

A workbook of 26 ISNCSCI cases with explanations for the correct classifications was developed by the authors to serve as an educational resource and training tool.^9^ The ISNCSCI workbook is freely available for download on the ASIA website^9^ and is also published in electronic form under an open access license in the Open Data Commons for Spinal Cord Injury (ODC-SCI).^13^ For this article, Cases 1 and 2 were selected from the workbook to (1) illustrate the documentation of AIS E grade and (2) provide an example of classification status post tendon transfer. Cases 3 through 5 were created separately from the workbook to address ISNCSCI classification in 3 specific scenarios: (1) spinal cord stimulation for neuromodulation, (2) nontraumatic SCI (ntSCI) etiology, and (3) AIS D grade (vs. AIS B) based on the presence of non-key muscle function. These scenarios were chosen because they relate to common areas of confusion, address challenging concepts, and have not previously been described. The cases were reviewed by members of the International Standards Committee to confirm agreement, and each response represents the committee's consensus. The 5 questions on ISNCSCI classification received from SCI professionals, the responses from the International Standards Committee, and illustrative cases are presented below.

## Questions and Responses

### Question 1

How are the classification components recorded on the ISNCSCI worksheet if a person presents with an ASIA Impairment Scale (AIS) E grade, with prior examinations demonstrating impairment (AIS A-D)?

### Response

The AIS E classification is intended for individuals with a history of SCI (e.g., previously documented neurological impairment on an earlier ISNCSCI exam) who experience improvement in sensorimotor function, resulting in an intact follow-up ISNCSCI exam. On the ISNCSCI worksheet, “INT” for “intact” is recorded in the boxes for sensory and motor levels and for the neurological level of injury (NLI). In cases of AIS E, examiners should document the previous NLI (preferably from the initial ISNCSCI exam) in the comments box, as this would provide valuable information about the injury and may help to explain and predict ongoing sequelae. For instance, an individual with AIS E grade and previous NLI of T1 may experience more autonomic dysfunction than a person with AIS E grade and previous NLI of T11. The International Standards Committee recommends that AIS E cases be considered neurologically incomplete if other neurological findings exist. Despite an intact ISNCSCI exam, a person with AIS E grade may have persistent neurological impairments and sequelae, including weakness in muscles not tested during the ISNCSCI exam, proprioception deficits, spasticity (hypertonia, spasms, dyssynergias, hyperreflexia, and pathological reflexes), autonomic dysfunction, and/or neuropathic pain. In such cases, these residual effects must be considered and evaluated as appropriate. For example, it is strongly recommended that the International Standards to document Autonomic Function following SCI (ISAFSCI)^14^ be used in conjunction with the ISNCSCI. The sensory and motor zones of partial preservation (ZPPs) are not applicable (NA) due to the presence of sacral sparing. See Case 1.

### Case 1: Classification of AIS E grade with no sensorimotor deficits detected on a follow-up ISNCSCI examination, with a previously documented SCI

**Figure:**
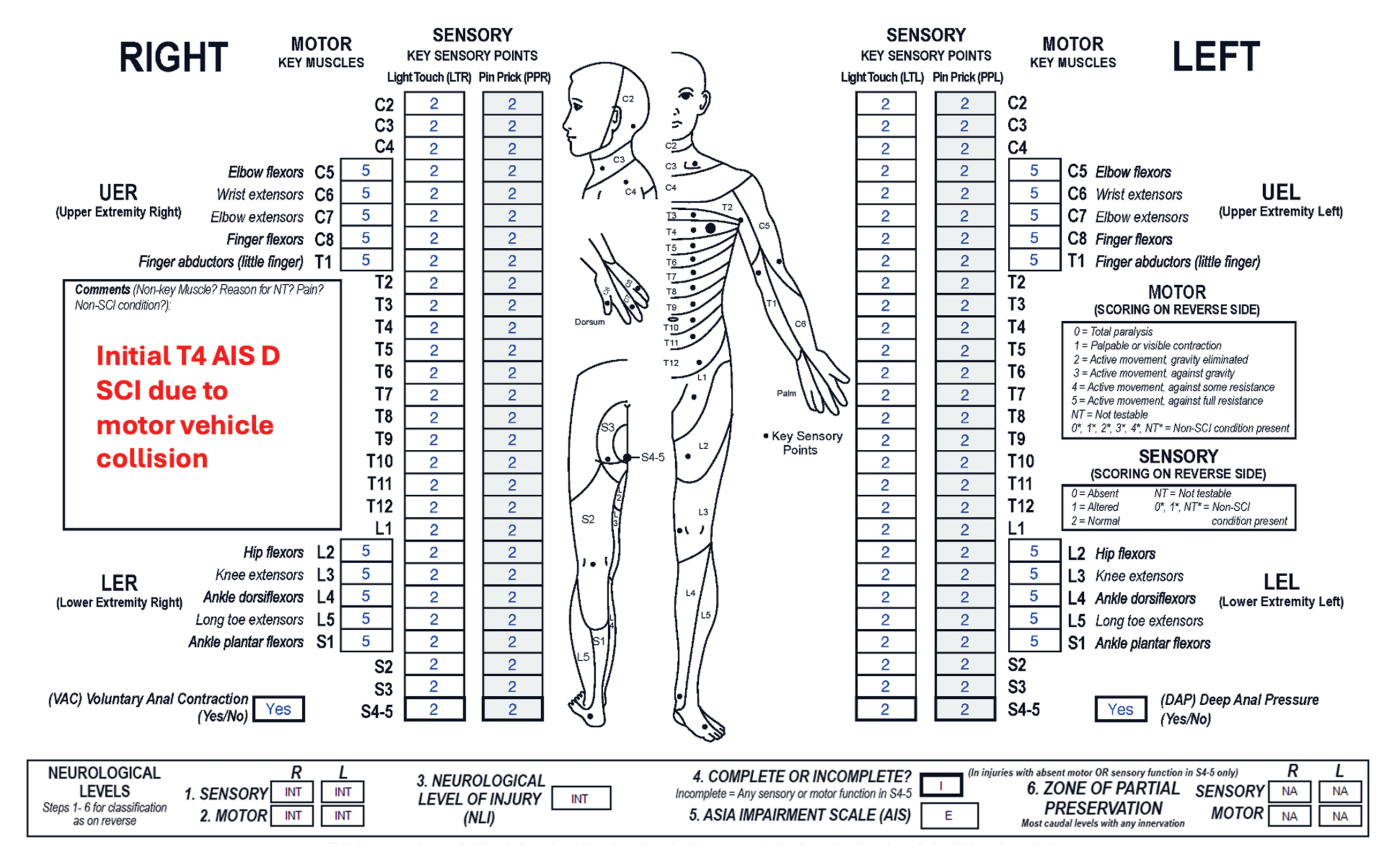


#### Sensory levels:

Light touch (LT) and pinprick (PP) sensation are intact throughout all dermatomes (including the most caudal sacral segments), so “INT” should be written in both sensory level boxes.

#### Motor levels:

Because motor function is intact in all key muscles and voluntary anal contraction (VAC) is preserved, “INT” should be recorded in the boxes for motor level.

#### Neurological level of injury (NLI):

“INT” should be recorded in the box for NLI. Note that an initial NLI of T4 is documented in the comments box.

#### Completeness:

This is considered an incomplete injury. Despite a normal ISNCSCI exam, individuals with AIS E injuries may have signs of persistent neurological impairment/sequelae, including weakness in muscles not tested with the ISNCSCI exam, spasticity, impaired proprioception, coordination deficits, gait disturbance, and autonomic dysfunction.

#### ASIA Impairment Scale (AIS):

The AIS grade is E because there are no longer any sensorimotor impairments detected with the components of the ISNCSCI exam. Note that an AIS E grade is only given if the individual previously had an SCI, in most cases based on an earlier ISNCSCI exam indicating some degree of sensorimotor impairment. If this case was a person's initial neurological exam without evidence of an SCI, there would be no classification performed and therefore no AIS grade assigned.

#### Sensory ZPPs:

The sensory ZPP is not applicable (NA) bilaterally because there is sensory sacral sparing on both sides; deep anal pressure (DAP) sensation is present, and both LT and PP sensation are preserved at bilateral S4-5 dermatomes.

#### Motor ZPPs:

The motor ZPP is NA bilaterally because voluntary anal contraction (VAC) is preserved.

#### Question 2

How do tendon and/or nerve transfers affect motor scores and classification components?

#### Response

The International Standards Committee recognizes the challenges and limitations of ISNCSCI classification in the setting of tendon and nerve transfers. A key principle of the ISNCSCI is that it is intended to characterize neurological function related to the spinal cord. Although there is emerging evidence that peripheral nerve and tendon transfers may induce central plasticity,^15^–^17^ it can be presumed, at least for classification purposes, that improvements in motor function following these procedures are primarily due to peripheral mechanics without significant changes in spinal segmental control. Individuals may receive a combination of nerve and tendon transfers, further confounding the clinical picture. It may be helpful to refer to the last ISNCSCI exam (prior to the surgical intervention) when performing the classification.

Case 2 (see below) represents a right posterior deltoid to triceps tendon transfer. Right elbow extension (C7 key muscle) is graded 3*. In this case, the tendon transfer is considered a non-SCI condition, and details of the upper extremity reconstruction are included in the comments box. As a general rule, if the strength of a key muscle function is impacted by reconstructive surgery, the examined motor grade requires an “*”. Within the comments box, it should also be specified how the “*”-tagged examined scores should be replaced by the assumed scores during classification. In this case, it can be assumed that if not for the restorative surgery, the recipient muscle (i.e., triceps) would not have strength sufficient for antigravity movements (i.e., motor grade <3 prior to the procedure), and this can be confirmed by referencing an earlier exam. It is also important to consider a case in which a key muscle (e.g., biceps brachii) is used as a donor. Although unlikely, this could weaken key muscle function, and in such cases, the examined motor score should be “*”-tagged. It should also be specified in the comments box how the “*”-tagged motor score is to be handled during classification (e.g., “considered normal” or “considered >4”). Again, referring to a presurgery ISNCSCI exam can be helpful. If no prior exam is available, it can be assumed that the donor muscle would have had a motor grade >4 prior to the procedure. The motor level should be “*”-tagged if its determination is impacted by a clinical assumption; other classification components may be similarly affected.

### Case 2: Classification of a cervical neurological complete (AIS A) injury in the setting of a tendon transfer

**Figure:**
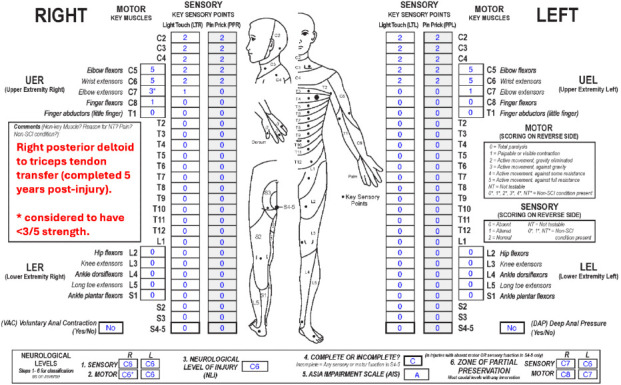


#### Sensory levels:

The sensory level is C6 bilaterally as this is the most caudal dermatome on both sides with intact sensory function, and sensation above this level is also intact.

#### Motor levels:

The right motor level is C6* because even though motor function is rated 3* at C7, the tested strength is impacted by a non-SCI condition, that is, a tendon transfer of the right posterior deltoid to the triceps. The tendon transfer does not affect the innervation to the triceps at the C7 segment. The motor grade at C7 without the transfer is presumed to be <3 (otherwise the tendon transfer would not have been indicated), so the right motor level is C6*; this requires an “*” because the designation is based on this clinical assumption. The left motor level is also C6 as this is the most caudal key muscle with a motor score >3, and all motor function rostral to this level is presumed to be intact.

#### NLI:

The NLI is C6 as each of the sensory levels and left motor level are C6. The right motor level is C6* based on clinical judgment (or would be C7 if strictly using the examined score). Therefore, the NLI does not require an “*” as it is not impacted by the non-SCI condition, which in this rare case leads to an improvement of key muscle function.

#### Completeness:

There is no sacral sparing, so this is a complete injury.

#### AIS:

The AIS grade is A as this is a complete injury.

#### Sensory ZPPs:

The sensory ZPP is applicable bilaterally because there is no sensory sacral sparing. The sensory ZPP is C7 on the right and C6 on the left, as these are the most caudal segments on the respective sides with any preserved sensory function.

#### Motor ZPPs:

The motor ZPP is applicable on both sides in the absence of VAC. The motor ZPP is C8 on the right and C7 on the left, as these are the most caudal myotomes on each side with any motor function. The motor ZPP on the right does not require an “*” because it is not impacted by the non-SCI condition in this particular case.

#### Question 3

If a person with a T9 AIS B SCI has volitional activation (≥1/5) of bilateral hip flexors (L2 myotome) only during transcutaneous spinal cord stimulation, does the injury classification change to AIS C?

#### Response

The role of the ISNCSCI in the setting of neuromodulation is a complex topic. First, there are different types of neuromodulation, including pharmacologic, electrical stimulation, and magnetic stimulation interventions; there is considerable variability in treatment effects, even among interventions of the same type. As a field, we are enhancing our understanding of these approaches, but there is much yet to be learned, such as each intervention's duration of effect and potential to induce neuroplasticity.

The ISNCSCI's fundamental purpose is to characterize neurological deficits resulting from a spinal cord lesion. Therefore, the International Standards Committee recommends that, when possible, the ISNCSCI examination be performed without the use of active neuromodulation interventions or neuroprostheses, such as spinal cord stimulation, functional electrical stimulation (FES), and brain-computer interfaces. In some cases, it may be more difficult to control for the effects of chemical neuromodulators depending on their half-life/duration of effect. Certain interventions may be continuous (e.g., intrathecal baclofen therapy) without the option to stop treatment for neurological testing. No matter the setting and scenario, one must understand the contextual factors when interpreting ISNCSCI results.

It is also important to mention that the ISNCSCI exam can be used by clinicians and researchers to characterize the effects of neuromodulation while it is active (and this active status must be documented). In such cases, it may not be appropriate to apply the classification as it would not accurately reflect the spinal cord function but rather the potentially transitory effects of the intervention. Even if neuromodulation, such as spinal cord stimulation, is turned on continuously in a person's daily life, an ISNCSCI exam performed without active neuromodulation (whenever possible) should be the standard for assessing and classifying the spinal cord lesion.

Regarding Question 3, if the motor grade at L2 is 0/5 in the absence of spinal cord stimulation, the AIS grade would remain B (see Case 3). Alternatively, if the neuromodulation resulted in lasting sensorimotor changes, and L2 motor function persisted while the spinal cord stimulator was turned off, the AIS grade would be C. Because the duration of carryover effects is not completely understood and may vary depending on the type of neuromodulation technique and protocol, researchers in clinical trials should document the temporal relationship between the intervention and collection of ISNCSCI data and specify this information in publications.

### Case 3: Classification of a thoracic AIS B injury after transcutaneous spinal cord stimulation

**Figure:**
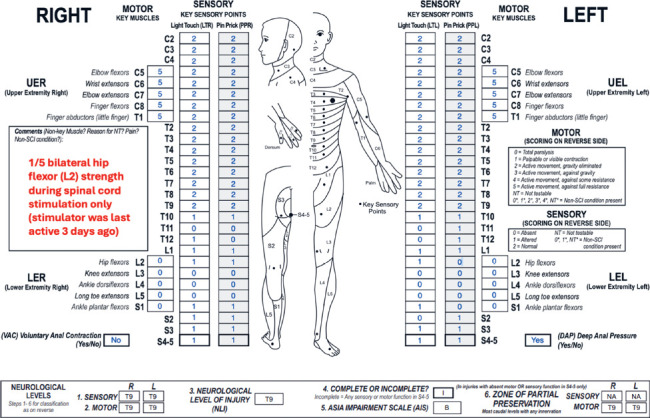


#### Sensory levels:

The sensory level is T9 bilaterally because sensory function is intact through this dermatome on both sides.

#### Motor levels:

Both motor levels defer to the sensory levels and are also T9. C5-T1 key muscles have full strength, and motor function is presumed to be intact through T9 based on the sensory scores.

#### NLI:

The NLI is T9 as each of the sensory and motor levels is T9.

#### Completeness:

This is an incomplete injury because there is sensory sacral sparing.

#### AIS:

The AIS grade is B as this is a sensory incomplete, motor complete injury. There is sensory sacral sparing as DAP and bilateral S4-5 LT/PP sensation are preserved. VAC is absent, and without spinal cord stimulation, there is no motor function more than 3 levels below the motor level on either side (this includes key muscles and non-key muscle functions). When determining the AIS grade, sensorimotor function that is present only in the setting of a neuroprosthesis or during neuromodulation therapy should not be considered. However, if L2 motor function of at least 1/5 strength persisted after neuromodulation, the AIS grade would be C as there would be sensory sacral sparing and preserved motor function more than 3 levels below the motor level (satisfying motor incomplete criteria).

#### Sensory ZPPs:

The sensory ZPP is NA bilaterally because there is sensory sacral sparing on both sides (DAP is present, and LT/PP sensation are preserved in the right/left S4-5 dermatome).

#### Motor ZPPs:

The motor ZPP is applicable bilaterally because VAC is absent, and it is T9 on both sides as there is no motor function below either motor level (in the absence of spinal cord stimulation). If there was preserved motor function (>1/5) at L2 bilaterally in the absence of spinal cord stimulation, both motor ZPPs would then be L2.

#### Question 4

Can the ISNCSCI be used in nontraumatic SCI (ntSCI)?

#### Response

The ISNCSCI can be used in ntSCI to help determine the level and severity of the neurological deficits resulting from the spinal cord lesion/disease. Degenerative myelopathy, spinal cord infarct, epidural abscess, spinal cord tumor, and transverse myelitis (see Case 4) are examples of ntSCI etiologies for which the full ISNCSCI classification is often appropriate. A recent study found the ISNCSCI to be valid and reliable in the setting of ntSCI.^18^ It is important to note that if there is generalized or multifocal spinal cord dysfunction, and if no single level of injury exists, the full ISNCSCI classification may not be determinable. For example, application of the classifications would not be suitable in cases of diffuse metastatic disease to the spine and multiple sclerosis with numerous brain and spinal cord lesions. Lastly, when using the ISNCSCI to predict neurological and functional recovery, it is important to understand that most of the literature on outcomes relates to traumatic SCI, and prognosis for persons with ntSCI may differ significantly. For example, a person with inflammatory myelopathy may experience rapid neurological recovery following treatment. Conversely, a person with metastatic cancer may experience precipitous deterioration due to tumor progression. There are some studies reporting neurological and functional outcomes in persons with ntSCI based on the initial ISNCSCI exam,^19^–^26^ but more research is needed.

### Case 4: Classification of an individual with ntSCI due to transverse myelitis with a single cervical lesion

**Figure:**
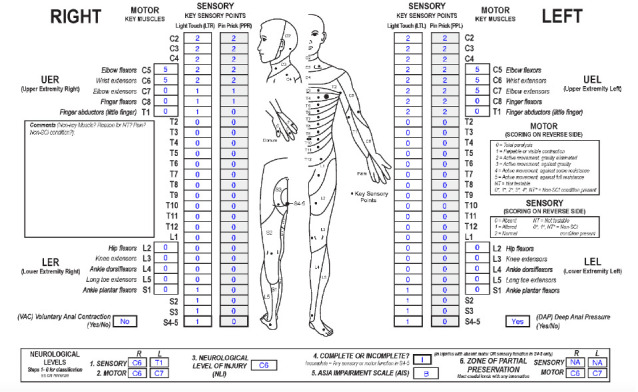


#### Sensory levels:

The sensory level is C6 on the right and T1 on the left because sensory function is intact through these dermatomes on the respective sides.

#### Motor levels:

The motor level is C6 on the right and C7 on the left as these are the most caudal key muscles on the respective sides with a grade ≥3, and all motor function above these segments is presumed to be intact.

#### NLI:

The NLI is C6 as this is the most rostral of the motor and sensory levels.

#### Completeness:

This is an incomplete injury because there is sensory sacral sparing.

#### AIS:

The AIS grade is B as this is a sensory incomplete, motor complete injury. There is sensory sacral sparing as DAP and bilateral S4-5 LT sensation are preserved. VAC is absent, and there is no motor function more than 3 levels below the motor level on either side (this includes key muscles and non-key muscle functions).

#### Sensory ZPPs:

The sensory ZPP is NA bilaterally because there is sensory sacral sparing on both sides (DAP is present and LT sensation is preserved in the right/left S4-5 dermatomes).

#### Motor ZPPs:

The motor ZPP is applicable bilaterally because VAC is absent, and it is C6 on the right and C7 on the left as there is no motor function below either motor level.

#### Question 5

Could the presence of non-key muscle function be used to determine an AIS B versus D injury?

#### Response

There are rare cases (particularly at lumbar injury levels) that meet criteria for AIS D grade, solely based on non-key muscle function, that would otherwise be classified as AIS B, if non-key muscle function was not present more than 3 levels below the motor level on either side (see Case 5). Non-key muscle function should be evaluated in all cases with sensory sacral sparing and absent VAC. Most often, the presence of non-key muscle function will be used to determine AIS B versus C grade.

### Case 5: Classification of AIS D grade based on preserved non-key muscle function

**Figure:**
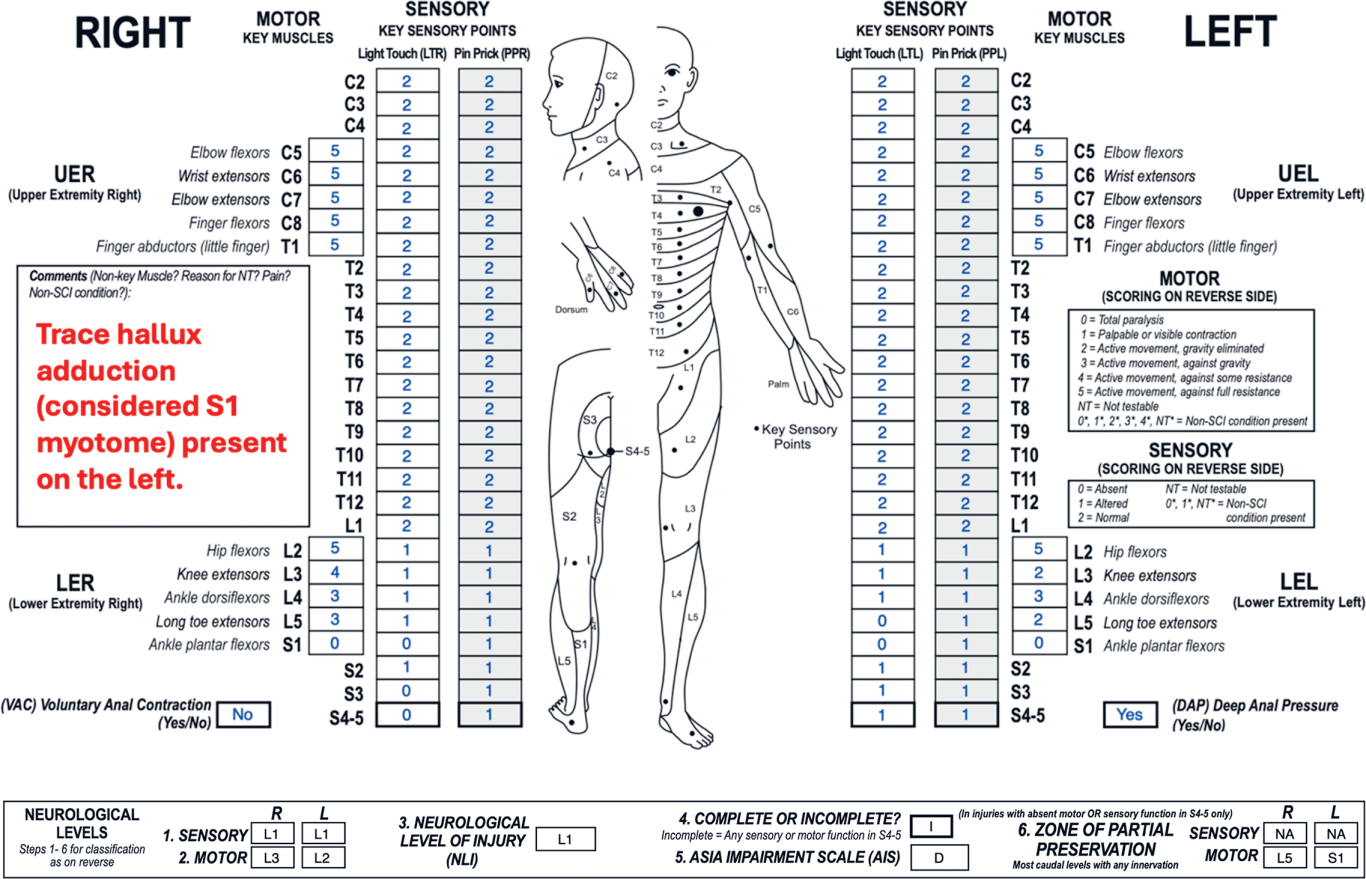


#### Sensory levels:

The sensory level is L1 bilaterally because sensory function is intact through this dermatome on both sides.

#### Motor levels:

The motor level is L3 on the right and L2 on the left as these are the most caudal key muscles on the respective sides with a grade ≥3, and all motor function above these segments is presumed to be intact.

#### NLI:

The NLI is L1 as this is the most rostral of the sensory and motor levels.

#### Completeness:

This is an incomplete injury because there is sensory sacral sparing.

#### AIS:

The AIS grade is D. Because DAP sensation is preserved (as well as right S4-5 PP sensation and left S4-5 PP/LT sensation), the lesion is at least sensory incomplete. VAC is absent, and there is no key muscle function more than 3 segments below the motor level on either side. However, this is an example of a scenario in which non-key muscle function should be tested to differentiate between AIS B and C/D. In this case, non-key muscle function is preserved in left hallux adduction (S1 myotome) as noted in the comments box. Therefore, this injury meets criteria for motor incomplete status as there is sensory sacral sparing and motor sparing at S1, which is more than 3 segments below the left motor level (L2). This injury is AIS D (and not AIS C) because at least half (in this case 6/10) of the key muscles below the NLI (L1) have a motor grade ≥3. Note that non-key muscle functions are not included in the calculation for AIS C versus D grade.

#### Sensory ZPPs:

The sensory ZPP is NA bilaterally because there is sensory sacral sparing on both sides.

#### Motor ZPPs:

The motor ZPP is applicable bilaterally because VAC is absent. On the right, it is L5 as this is the most caudal segment on that side with partially preserved motor function. The left motor ZPP is S1; while non-key muscle functions are not typically included in the determination process of motor ZPPs, this is a unique scenario in which the presence of a non-key muscle function is the only motor finding that defines the case as motor incomplete, so the associated myotome (S1) is recorded as the left motor ZPP.

## Discussion

The 5 questions/responses and cases presented in this article reinforce key objectives of the ISNCSCI, which are to characterize sensorimotor impairments directly resulting from a spinal cord lesion and to provide a comprehensive classification that includes injury level, severity, and partially preserved segments. As clinical practice and research in SCI medicine advance and interventions to restore function are utilized (e.g., surgical reconstruction of the upper extremity and application of neuromodulation/neuroprostheses), one must consider the ISNCSCI's purpose as well as its limitations.

The first case presented in this article reviews the classification of an AIS E injury. The reported percentage of persons who improve to AIS E grade varies widely within the literature.^27^,^28^ Differences in the timing of baseline and follow-up assessments, initial AIS grade, level of injury, and databases used are some factors contributing to this large variation. In general, 0% to 33% of persons with initial AIS D grade will improve to AIS E by one-year post injury.^27^ It is important for clinicians, researchers, and those with SCI to recognize that even after neurological recovery to an AIS E classification, neurological sequelae unmeasured by the ISNCSCI examination may persist.^27^ This could include, but is not limited to, autonomic dysfunction, neurogenic bladder/bowel, weakness in muscles not tested as part of the ISNCSCI exam, spasticity, impaired proprioception, coordination deficits, gait disturbance, and neuropathic pain, and may continue to impact one's function, participation, and quality of life.

Case 2 provides an example of ISNCSCI classification following a tendon transfer. With the 2019 ISNCSCI update, the new taxonomy for non-SCI-related conditions was introduced. Although not initially designed for such a scenario, a tendon transfer case can now be handled in a systematic way.^2^,^5^ This revised system incorporates clinical judgment and allows for a more accurate assessment of spinal cord function. Despite this increased utility, there are times when it may be too challenging to distinguish SCI-related neurological{pg=} changes from those of a non-SCI condition (e.g., following nerve transfer surgery, especially if performed early after injury). As such, particularly in clinical practice, it may be helpful to refer to a previous ISNCSCI examination that was performed prior to the surgical intervention.

In Case 3, a complex issue regarding the ISNCSCI and neuromodulation is introduced. In general, the International Standards Committee recommends that the ISNCSCI examination be performed without active neuromodulation or the use of neuroprostheses to more accurately assess residual spinal cord function. Therefore, the ISNCSCI classification should not change if sensorimotor effects are transitory and only present during a particular intervention. This also calls into question the duration of carryover effects. For example, would there be a difference in one's ISNCSCI examination if performed 30 minutes after spinal cord stimulation versus 30 days post intervention? The International Standards Committee recognizes that this will be an ongoing discussion and will require further research. For now, it is recommended that the temporal relationship between neuromodulation therapy and acquisition of ISNCSCI data be recorded and described in publications. Furthermore, there is evidence that neuromodulation, such as spinal cord stimulation, may lead to long-term improvements in sensorimotor function,^29^ and these lasting changes should be reflected in the ISNCSCI examination and classification.

The fourth case provides an example of ISNCSCI classification in someone with an ntSCI. For traumatic SCI, the ISNCSCI's psychometric properties have been thoroughly studied, and it has been shown to be a highly valid, reliable, and responsive instrument.^28^,^30^ More recently, the ISNCSCI has been found to be a valid and reliable tool in the neurological evaluation of persons with ntSCI.^18^ The ISNCSCI can be used to document and characterize sensorimotor impairments in this clinical population, especially when there is a focal spinal cord lesion. The applicability of ISNCSCI classifications is less suitable for conditions such as multiple sclerosis with widespread neuraxis involvement, hereditary spastic paraplegia with progressive degeneration of the long tracts, and diffuse metastatic disease.

Knowing when and how best to utilize the ISNCSCI for those with ntSCI is essential, in both the clinical and research settings. The incidence of ntSCI is increasing,^31^–^34^ and in some centers it exceeds that of traumatic SCI. Several SCI databases, such as the Rick Hansen Spinal Cord Injury Registry (RHSCIR), New Zealand Spinal Cord Injury Registry (NZSCIR), US Department of Veterans Health Administration Spinal Cord Injuries/Dysfunction Registry (VHA SCIDR), Nordic Spinal Cord Injury Registry (NordicSCIR), Germany-wide web-based ParaReg registry, and National Spinal Cord Injury Model Systems (SCIMS), are now including individuals with ntSCI (some with exclusion of specific etiologies such as multiple sclerosis and amyotrophic lateral sclerosis). Additionally, the International SCI Core Data Set includes ISNCSCI variables, and its use has been recommended for persons with ntSCI.^35^

Finally, Case 5 addresses the role of non-key muscle function in the determination of motor incomplete status. This challenging concept was first introduced in 2003^36^ and was clarified in 2011.^37^ Case 5 demonstrates an especially rare scenario of AIS B versus D grade that has not previously been described in the literature. Understanding when to test non-key muscle functions is important because results may greatly impact the classification, as seen in this case. Please note that the most common nonkey muscle functions and associated myotomes can be found on the back of the ISNCSCI worksheet. It is also important to mention that most SCI registries do not include data on non-key muscle function.

The International Standards Committee appreciates feedback and questions from colleagues within the field. Such input has influenced prior ISNCSCI revisions and will similarly contribute to future updates. The International Standards Committee hopes that these cases, responses, and review of the correct classifications, as well as the full ISNCSCI workbook^9^ and International Standards Training e-Learning Program (InSTeP),^38^ will serve as valuable resources for those interested in enhancing their classification skills. Furthermore, scenarios similar to those described in this article are becoming increasingly more common in practice. Each response represents a consensus from the International Standards Committee on how best to classify such cases.

## Conclusion

The International Standards Committee provided answers to questions on ISNCSCI classification. Five case examples related to AIS E grade, tendon transfers, spinal cord stimulation, nontraumatic SCI, and AIS B versus D grade (based on non-key muscle function) were presented to reinforce classification rules and committee recommendations. This article can serve as a useful reference when similar cases are encountered in clinical and research settings.
